# Perfluorocarbon emulsions radiosensitise brain tumors in carbogen breathing mice with orthotopic GL261 gliomas

**DOI:** 10.1371/journal.pone.0184250

**Published:** 2017-09-05

**Authors:** Lisa A. Feldman, Marie-Sophie Fabre, Carole Grasso, Dana Reid, William C. Broaddus, Gregory M. Lanza, Bruce D. Spiess, Joel R. Garbow, Melanie J. McConnell, Patries M. Herst

**Affiliations:** 1 Department of Neurosurgery, Virginia Commonwealth University, Richmond, VA United States of America; 2 Malaghan Institute of Medical Research, Wellington, New Zealand; 3 School of Biological Sciences, Victoria University, Wellington, New Zealand; 4 Division of Cardiovascular Diseases, Washington University School of Medicine, St. Louis, MO United States of America; 5 Department of Anesthesiology, College of Medicine, University of Florida, Gainesville, FL United States of America; 6 Mallinckrodt Institute, Washington University School of Medicine, St. Louis, MO United States of America; 7 Department of Radiation Therapy, University of Otago, Wellington, New Zealand; Swedish Neuroscience Institute, UNITED STATES

## Abstract

**Background:**

Tumour hypoxia limits the effectiveness of radiation therapy. Delivering normobaric or hyperbaric oxygen therapy elevates pO_2_ in both tumour and normal brain tissue. However, pO2 levels return to baseline within 15 minutes of stopping therapy.

**Aim:**

To investigate the effect of perfluorocarbon (PFC) emulsions on hypoxia in subcutaneous and intracranial mouse gliomas and their radiosensitising effect in orthotopic gliomas in mice breathing carbogen (95%O_2_ and 5%CO_2_).

**Results:**

PFC emulsions completely abrogated hypoxia in both subcutaneous and intracranial GL261 models and conferred a significant survival advantage orthotopically (Mantel Cox: p = 0.048) in carbogen breathing mice injected intravenously (IV) with PFC emulsions before radiation *versus* mice receiving radiation alone. Carbogen alone decreased hypoxia levels substantially and conferred a smaller but not statistically significant survival advantage over and above radiation alone.

**Conclusion:**

IV injections of PFC emulsions followed by 1h carbogen breathing, radiosensitises GL261 intracranial tumors.

## Introduction

Despite aggressive treatment with surgery, temozolomide chemotherapy and radiation therapy, median survival of glioblastoma (GBM) patients after initial diagnosis averages only 15–20 months [1–3]. The addition of targeted therapies such as bevacizumab, sunitinib, gefitinib, erlotinib and irinotecan, has failed to increase overall survival beyond 23 months (reviewed in [4]). Cancer-specific radiosensitisation by removing tumor hypoxia is a promising strategy for improving patient survival and quality of life. Although GBMs are highly vascularized, their blood supply is compromised as blood vessels are tortuous and leaky with microvascular hyperplasia, leading to transient areas of hypoxia and thus radiation resistance [5]. Radiation damages DNA either directly or indirectly by producing reactive oxygen species (ROS) in the vicinity of DNA. Under hypoxic conditions the ROS-generated DNA backbone lesions are easily repaired. Oxygen converts these repairable DNA lesions to permanent lesions [6,7] and destabilises the hypoxia-regulated master switch, HIF-1α. Loss of HIF-1α results in switching from a highly invasive phenotype, associated with a glycolytic metabolism, increase in glioma stem cell marker expression and treatment resistance, to a less invasive phenotype that relies on mitochondria as an energy source [8–13]. In addition, hyperoxia can re-sensitise chemoresistant GBM cells to temozolomide [14], promote infiltration of tumor-specific CD8 T cells and decrease regulatory T cell activity [15].

The impact of hypoxia and its modification on the outcome of radiation has recently been reviewed by Horsman and Overgaard [16]. The highly invasive nature of GBMs means that irradiating normal brain tissue is unavoidable and makes tumor specific oxygenation challenging. In this paper we attempted to achieve this by injecting mice IV with perfluorocarbon (PFC) emulsions. PFC emulsions are chemically inert nanoparticles (<0.2μm diameter) comprised of carbon chains substituted with halogens (fluorine atoms). By fluoridating the carbons and removing all hydrogens the polar hydrocarbon oils become, non-polar inert fluids of varying viscosity. These non-polar liquids demonstrate very high gas solubility coefficients for non-polar respiratory gases (i.e. O_2_, N_2_ and CO_2_). As such the dissolved gas content of PFCs is dependent upon the specific carbon compound utilized as the parent molecule for complete fluoride substitution and the partial pressure of the particular gas in its environment. All respiratory gases move easily through PFCs and all molecules held dissolved in the liquid are available for biologic activity (unlike hemoglobin binding of oxygen). PFCs enhance oxygen diffusion in between erythrocytes and target tissues by removing/reducing the barrier of polar aqueous fluid- plasma. Therefore the enhanced diffusion of gases has been measured at 10–50 fold increased if PFCs are present in whole blood [17]**.** Unlike haemoglobin, PFCs show enhanced non-polar gas solubility (oxygen) as compared to water based liquids (plasma). The enhanced solubility allows them to release oxygen rapidly under low PO_2_ in a linear manner [18], and making all oxygen available for metabolism, thereby delivering more oxygen to hypoxic tumors than to normoxic normal brain tissue and conferring a degree of cancer-specificity. PFCs are maximally loaded with oxygen when the subject breathes 100% oxygen after intravenous injection [19,20]. However in models of brain injury 50% inspired oxygen seemed as efficacious as 100% oxygen. PFC nanoparticles are phagocytosed by the mononuclear phagocyte system, which slowly releases them back into the bloodstream attached to blood lipids. In the lungs PFCs pick up oxygen to deliver to tissues; they are cleared predominantly in the liver and spleen and via the bile and intestines in rodents [18]. Phase I and II clinical trials in patients with primary high-grade gliomas showed that IV administration of the first generation PFC emulsion, Fluosol, along with short-term oxygen exposure had a strong safety profile and produced a small (statistically insignificant) radiosensitising effect [21]. The second generation PFC emulsion, Oxygent, with more PFCs and a longer intravascular half-life [18] was shown to radiosensitise Lewis lung tumors in carbogen (95%O_2,_ 5%CO_2_) breathing mice [22], improve cognitive recovery after traumatic brain injury in rats [23] and has been used successfully as a substitute for blood transfusions during surgery [20,24]. Rockwell and colleagues explored the use of Fluosol [25] and Oxygent [26] in combination with hyperbaric oxygen (HBO) as adjuvants to radiation therapy. The authors showed that the PFC emulsion/HBO combination increased the radiation sensitivity of rat rhabdomyosarcoma BA1112 tumors in WAG/rij-Y rats relative to normal tissues, thereby enhancing the therapeutic ratio. In this paper, we describe the effects of a third-generation PFC emulsion similar to Oxygent in combination with carbogen breathing on hypoxia and tumor progression, after radiation, in the intracranial GL261 mouse glioma model.

## Material and methods

### Materials

Unless otherwise noted, tissue plasticware was purchased from Corning (In Vitro Technologies, Auckland, New Zealand); all cell-culture reagents were from Gibco BRL (Thermo Fisher Scientific, Auckland, New Zealand). All other chemicals and reagents were from Sigma Chemical Company (St. Louis, MO., U.S.A.). Hydroxyprobe TM-1 Plus kit containing pimonidazole hydrochloride, FITC- conjugated mouse IgG1 monoclonal; antibody (FITC-MAB1) and rabbit anti-FITC conjugated with horseradish peroxidase were from Hydroxyprobe Inc (Burlington, MA, USA). PFC emulsions were kindly provided by Professor Gregory M Lanza (Washington University School of Medicine, St. Louis, MO USA). The PFC emulsions (8ml) consisted of 40% PFC nanoparticles (3.2mL) suspended through sonification (20,000 psi for 5 passes) in 4% lecithin (0.32mL), water (4.37mL) and glycerine (0.11mL). Nanoparticles were 229.4 nm in size with a polydispersity of 0.055 and a zeta potential of -18.2 mV. The biological half-life of the PFCs is 3.5 days.

### Cell lines

The mouse glioma cell line, GL261 was obtained from the NCI tumor-cell-line repository (Frederick, MD, USA). Mycoplasma-free GL261 cells were grown in DMEM supplemented with 10% (v/v) FBS, GlutaMAX-1 (2mM), and maintained in a humidified incubator at 37°C/5% CO_2_.

### Mouse models

All experiments using mice were conducted in accordance with the New Zealand Animal Welfare Act 1999 and were approved by the Animal Ethics Committee of Victoria University, Wellington (NZ) (protocol number 22333).

#### Subcutaneous mouse model

Live mouse glioma GL261 cells (5X10^6^) were injected subcutaneously in 0.1ml of DMEM into the flanks of 8–12 week old (approx. 30g) male C57BL/6 mice. Tumor growth was measured every other day using electronic calipers. Tumor volume was calculated using the formula (length X width^2^)/2, where length represents the longest axis and width was measured at right angles to length. Animals were sacrificed by CO_2_ inhalation when the tumor volume reached 1000 mm^3^ or upon ulceration, whichever occurred first.

#### Intracranial mouse model

Live mouse glioma GL261 cells (25 x 10^3^ in 2μl of PBS) were implanted into the brains of 8–12 week old (approximately 30g) male C57BL/6 mice as described previously [27]. Animals were anaesthetized by i.p. injection of xylazine (100mg/kg) and ketamine (10mg/kg), (Phoenix Pharm), and Lacri-Lube (Allergan) was applied across the corneas of the eyes. A burr hole was drilled in the skull 0.1mm posterior to the bregma and 2.3mm lateral to the midline. Cells were administered stereotactically (Stoelting Apparatus), through the burr hole into the right putamen, using a Hamilton syringe with a 32-gauge needle. The needle was advanced to a depth of 2.3mm from the brain surface and the cell suspension delivered slowly over the course of 2 to 3min. Following injection, the needle was left in place for 2min, after which time, it was raised to a depth of 1.5mm and left in place for an additional 1min. The burr hole was sealed with bone wax and the incision sutured. Animals received sub-cutaneous analgesics (Carprofen (5mg/kg), Norbrook Laboratories, and Buprenorphine (0.1mg/kg), Renckitt Benckiser Pharmaceuticals), to control post-operative pain. Animals were randomly assigned into control and treatment groups (n = 4-5/group). Animals were weighed daily and humanely sacrificed by CO_2_ inhalation when they lost >10% body weight or when neurological signs of disease were evident, whichever occurred first. We were unable to image the brain to confirm the presence of tumour before treatments.

### Whole brain irradiation of mice

**Fig 1:**
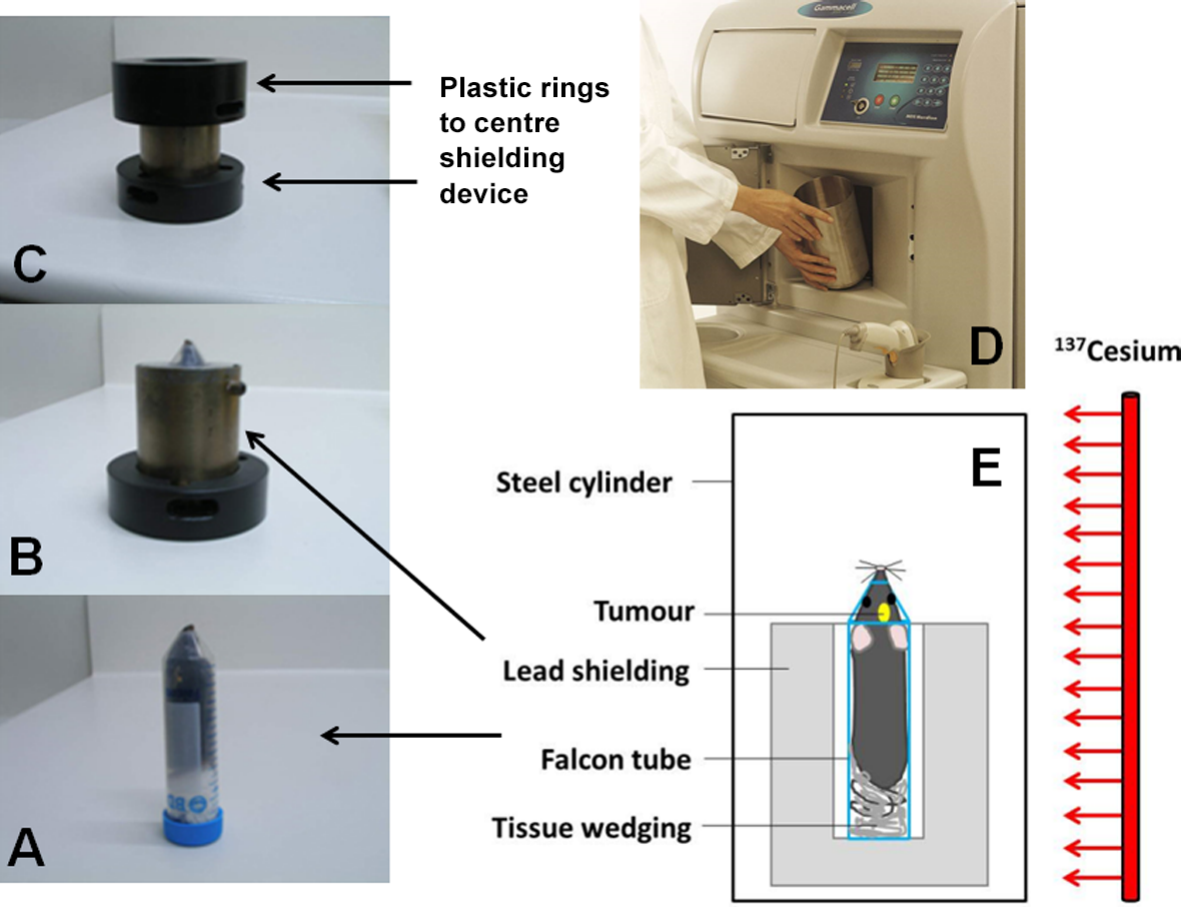
Irradiation setup for whole brain irradiation of mice. (A) The mouse is anaesthetized and positioned in a Falcon tube (B) inside a 2 cm thick lead shielding device (C) inside plastic rings (D) inside the aluminium cylinder in the Gammacell 3000 Elan irradiator. (E) Diagrammatic representation of the irradiation setup. This setup allowed us only to do whole brain irradiation; we could not irradiate subcutaneous tumors.

Mice received a single dose of 4.5Gy to the brain (and ≤1 Gy to the shielded body) on different days after surgery using a Gammacell 3000 Elan irradiator (Best Theratronics), which irradiates the content of a steel cylinder with γ-rays from a sealed Cesium-137 line source as described previously [28]. Briefly, tumor-bearing mice were anaesthetized with ketamine/xylazine (100/15mg/kg) and placed in an upright position in a 50mL Falcon tube without a tip to facilitate breathing. The tube was placed inside 2cm thick custom-built lead shielding that exposes the head to radiation whilst shielding the body from the ears down (see Fig 1).

### Treatment of mice with PFC emulsions and carbogen

Tumor bearing mice received tail vein IV injections with 1.5cc/kg of a 40% PFC emulsion (400μl per mouse). PFC injected mice were pre-treated with a single intraperitoneal injection of Carprofen (5mg/kg) per mouse to counter a general inflammatory response to the PFCs. Mice that received carbogen (95%O_2_/5%CO_2_) for 1h were placed in an air-tight chamber. Carbogen gas was delivered at a low flow rate from a premixed cylinder (BOC Gas & Gear, Wellington, New Zealand) via a tube through a small hole in the chamber. Oxygen levels were maintained at 85–95% and carbon dioxide levels at 4–5% (measured by a Gas analyzer ML206, ADInstruments, Colorado Springs, CO, USA).

### Brain and subcutaneous tumor tissue collection

At the experimental endpoints, animals were euthanised, whole brains were rapidly harvested, snap-frozen in liquid nitrogen using a Gentle Jane® snapfreezer (Instrumedics Inc, New Jersey, USA) and cryosectioned. For intracranial tumors, whole body perfusion was performed with PBS, following the method as described in [29].

### Hypoxia detection

Pimonidazole-HCl (PIM) was used to detect hypoxia. PIM binds irreversibly to thiol groups in proteins, peptides and amino acids in the cytoplasm of hypoxic cells and gives an accurate estimate of radiobiologically relevant hypoxia [30]. PIM was administered *via* intraperitoneal injection at a dosage of 60mg/kg body weight and 1h later brains and subcutaneous tumors were snap-frozen and cryosectioned. Sections were fixed for 2min in icecold acetone, rehydrated in PBS+0.1% Tween 20 and blocked with rabbit serum diluted 1/200 in PBS. Sections were rinsed 3x for 5min in PBS. For detection of PIM, sections were incubated with FITC anti-mouse IgG1 (FITC-Mab1 from Hydroxyprobe ™-1 Plus Kit,) diluted 1:200 in PBS, overnight at 4°C and incubated for 1h with rabbit-anti-FITC conjugated with horseradish peroxidase and diluted 1:200 in PBS. Slides were washed in PBS, dried and mounted with DAPI containing mounting media (ProLong Gold Antifade, Thermo Fisher Scientific, Auckland, New Zealand). Fluorescently labelled slides were imaged using a confocal fluorescent microscope (Olympus IX83; FV 1200 Tokyo, Japan).

### Statistical analysis

The Mantel-Cox log-rank test (Prism 5.0 Graph Pad Software, Inc. La Jolla, CA, USA) was used to determine statistical significances between Kaplan-Meier survival curves of the different groups indicated in Table 1. In all instances, p<0.05 was considered statistically significant.

**Table 1:** Statistical data for Fig 5.

Groups	n	Mean (days)	Range (days)	p-value (Mantel Cox test) (for difference between groups)
**Control**	13	24	20–29	0.139 (C and RT)
**RT**	9	26	23–29	0.243 (RT and RT/C)
**RT/C**	12	27	22–37	0.288 (RT/C and RT/C/PFC)
**RT/C/PFC**	9	30	22–39	**0.48 (RT and RT/C/PFC)**

## Results

### PFC emulsions and carbogen abrogate tumor hypoxia in a subcutaneous model

**Fig 2:**
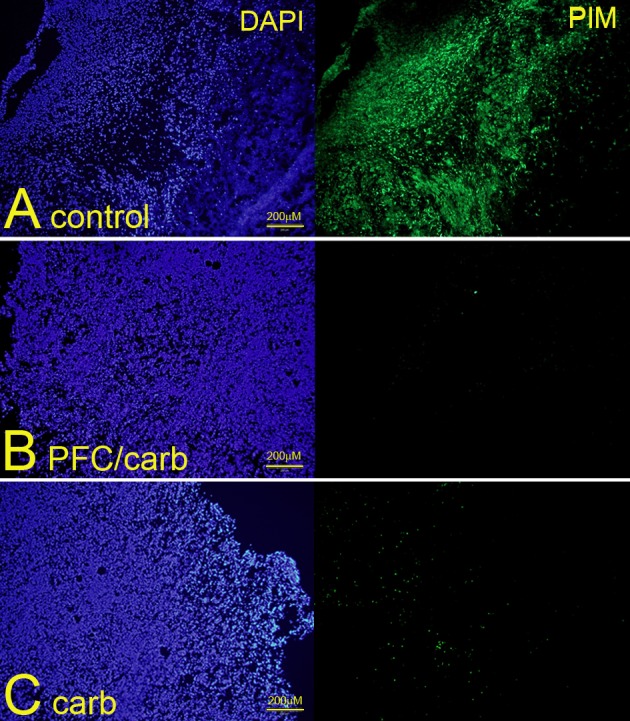
Effect of PFC/carbogen and carbogen alone on hypoxia in subcutaneous mouse glioma tumours. Animals were divided into groups of 5 mice each. (A) Untreated control mice; (B) mice exposed to both PFC emulsions and carbogen and (C) mice exposed to carbogen alone. Tumor-bearing mice were injected IV with 1.5cc/kg of a 40% PFC emulsion and hypoxia was measured using pimonidazole-HCL (PIM). Mice that received carbogen (95%O_2_/5%CO_2_) for 1h were placed in an air-tight chamber. Photos are representative of 2 separate experiments with 5 mice in each group.

Animals bearing small subcutaneous tumors were divided into three groups (n = 5 each); one group was injected with PFC emulsions and exposed to carbogen for 1h, a second group was exposed to carbogen for 1h and a third control group breathed normal air throughout the experiment. Fig 2 shows that PFC/carbogen completely abrogated tumor hypoxia, whilst carbogen alone substantially reduced hypoxia in tumors. Because of the irradiation set-up in our laboratory (Fig 1) we were unable to irradiate subcutaneous tumours. We therefore completed the rest of the experiments in the intracranial model.

### PFC emulsions and carbogen abrogate tumor hypoxia in an intracranial model

**Fig 3:**
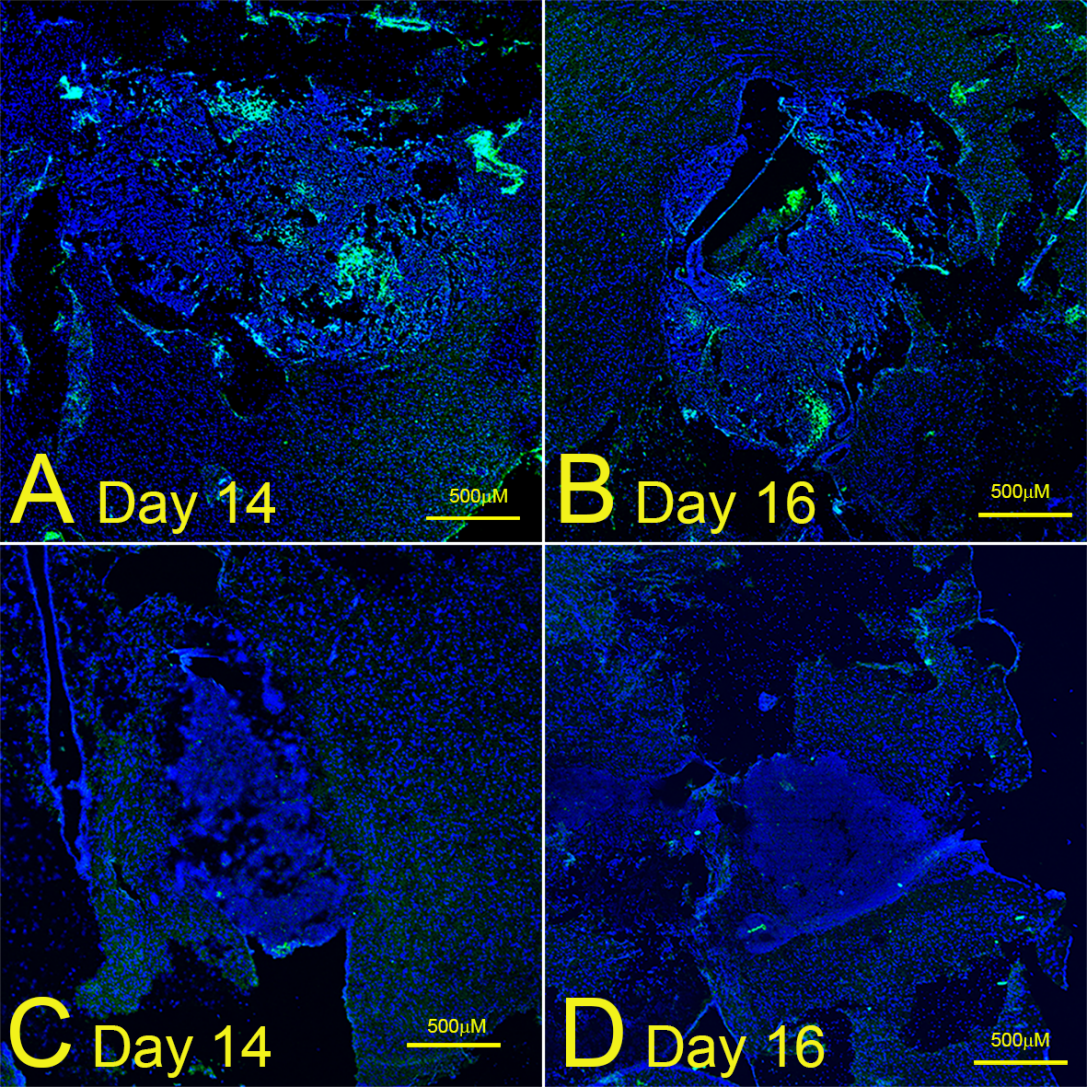
Different hypoxia levels in intracranial tumours of untreated control mice in the same experiment. **(**A) and (C): day 14 after implantation; (B) and (D): day 16 after implantation. Photographs are from one experiment with 4 mice (A-D).

**Fig 4:**
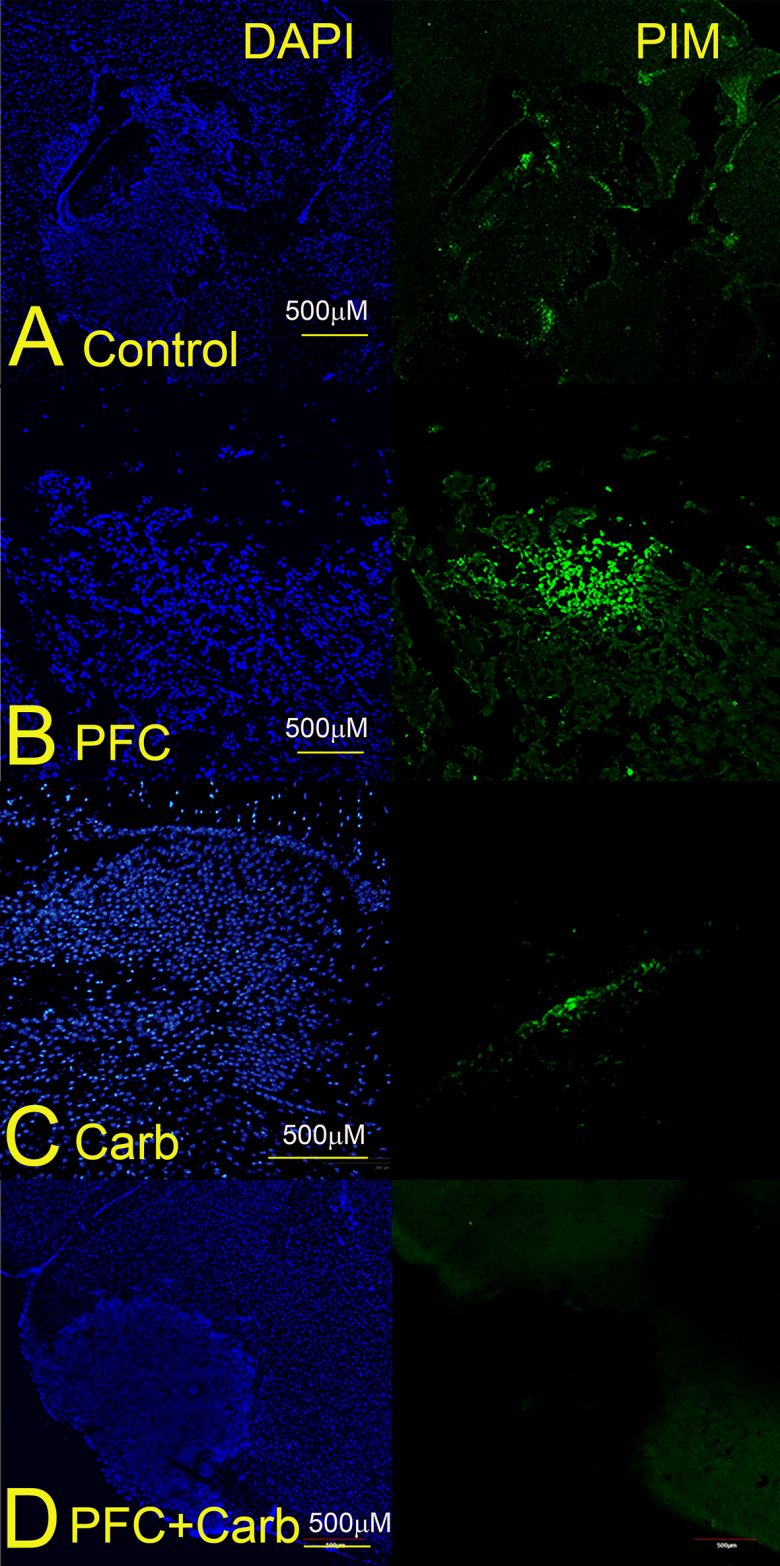
Effect of PFC/carbogen and carbogen alone on hypoxia in intracranial mouse glioma tumours. Animals were divided into groups of 5 mice each. A. Untreated control mice; B. Mice exposed to PFC emulsions; C. Mice exposed to carbogen and D. Mice exposed to both PFC emulsions and carbogen. Tumor-bearing mice were injected IV with 1.5cc/kg of a 40% PFC emulsion and hypoxia was measured using pimonidazole-HCL (PIM). Mice that received carbogen (95%O_2_/5%CO_2_) for 1h were placed in an air-tight chamber. Photos are representative of 3 separate experiments.

We repeated the hypoxia experiments in our intracranial model, where we irradiated the whole brain of tumor-bearing mice with a single dose of 4.5Gy a set number of days after implantation. In In contrast to the large areas of hypoxia in the subcutaneous model, only 2 out of 4 intracranial tumours in one experiment (Fig 3) and 3 out of 5 intracranial tumours in a second experiment (results not shown) displayed small and patchy areas of hypoxia 14–22 days post-implantation. Tumor size was unrelated to the number or size of the hypoxic patches. Hypoxia was completely abrogated in tumors exposed to both PFC/carbogen, whereas carbogen alone decreased the level of hypoxia, but did not remove it completely whereas exposure to PFCs alone did not affect hypoxia levels (Fig 4).

### PFC emulsions and carbogen radiosensitise GBM tumours in an intracranial model

**Fig 5:**
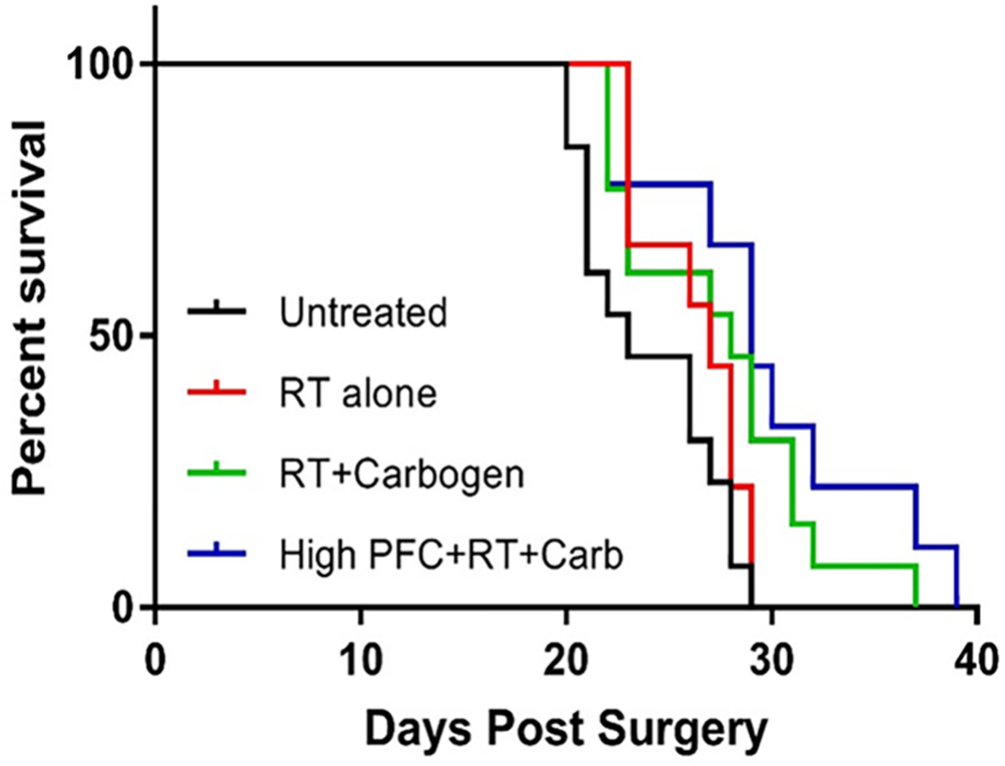
The effect of PFC plus carbogen and carbogen alone on survival of mice with intracranial tumours who received a single dose of whole brain radiation. Kaplan Meier curves were compiled from 3 separate experiments. Mice were randomly assigned to groups of 5 mice. Mice were left untreated (black line), given 4.5Gy of radiation (red line), breathing carbogen (95%O_2_/5%CO_2_) for 1h immediately prior to irradiation (green line) or injected IV with 1.5cc/kg of a 40% PFC emulsion in addition to breathing carbogen for one hour immediately prior to irradiation (blue line). Only data from animals that died from tumour progression were used. Data from animals that died from other causes were excluded.

Our final and clinically most interesting set of experiments investigated the effect of PFC/carbogen, and carbogen alone on radiosensitisation on day 18 post-implantation. As can be seen in Fig 5 and Table 1, PFC plus carbogen radiosensitised intracranial GL261 tumors, resulting in significantly longer survival in these animals over animals treated with radiation alone (p = 0.048). There was a similar trend towards longer survival in animals treated with carbogen alone, but this difference was not statistically significant (p = 0.288).

## Discussion

Based on earlier studies [20,21,23,24], we hypothesized that a third generation PFC emulsion similar to Oxygent would 1] decrease hypoxia and 2] radiosensitise GBMs in carbogen breathing mice. Increasing oxygen levels in GBM tumors should improve clinical outcomes by increasing the indirect (free radical-) effect of radiation, producing a less invasive phenotype, improving tumor response to temozolomide and stimulating an anti-tumor immune response. In this paper, we investigated whether or not a third-generation PFC emulsion would decrease hypoxia and radiosensitise intracranial GL261 tumors in carbogen-breathing mice. We found that 1] PFCs completely abrogated tumor hypoxia in both subcutaneous and intracranial GL261 glioma models in carbogen-breathing mice and that 2] PFC/carbogen-treated mice lived significantly longer after receiving whole-brain radiation, compared to mice treated with radiation alone. PFC emulsions alone did not affect tumor hypoxia, demonstrating the importance of high oxygen exposure to enable the PFCs to deliver sufficient oxygen [19,20]. Carbogen breathing alone was successful in reducing hypoxia in both subcutaneous and intracranial GL261 models, but failed to demonstrate a statistically significant survival advantage over and above irradiated mice. Because we could not irradiate subcutaneous tumors in our irradiation set-up, we were unable to test the effect of PFC emulsions on tumour growth after exposure to a range of radiation doses. Similarly we were unable to extend the dose to the intracranial tumors significantly because we were only able to irradiate the whole brain. We were therefore unable to calculate the oxygen enhancement ratio. We did however irradiate intracranial tumours of different sizes (between day 14 and 22 after implantation) and found no difference in the extent of hypoxia between large and small tumours (see Fig 3).

Two stage III clinical trials combined two hypoxia-reducing treatments. The BCON (Bladder, CarbOgen, and Nicotinamide) trial co-administered carbogen (98%O_2_ + 2% CO_2_) to decrease diffusion-limited hypoxia, and vasoactive nicotinamide to limit hypoxia caused by intermittent reduction of blood flow in tumors [31]. The BCON trial reported that radiation with concurrent carbogen and nicotinamide conferred a significant benefit in 5-year overall survival for patients on radiotherapy/carbogen/nicotinamide compared to patients on the radiation therapy arm alone (50% and 39% respectively) in 333 patients with locally advanced bladder cancer. Late morbidity was similar in both trial arms [31]. The ARCON (Accelerated Radiation, CarbOgen, and Nicotinamide) trial randomly assigned 345 patients with cT2-4 laryngeal cancer to either AR or ARCON and reported a significant gain in regional control rate for ARCON compared with AR, with similar acute and late toxicities [32]. Hyperbaric oxygen therapy within 15min of radiation therapy has also been reported to be safe and deemed effective in patients with high-grade gliomas [33,34]. Similar to normobaric carbogen treatment, increased pO_2_ values after hyperbaric oxygen therapy were only maintained for approximately 15min after returning to normal atmospheric pressure [33,35]. Interestingly, Clarke and colleagues recently showed that the time-frame for hyperoxic radiosensitisation may be much longer than commonly accepted and is independent of the presence of oxygen at the time of irradiation [36]. The pO_2_ of intracranial U87 tumors dropped back to baseline levels in mice breathing normal air for 25min after a pre-treatment with 100% O_2_ for 25min. However, O_2_ pre-treatment still conferred a statistically significant radiosensitisation effect. This transient radiosensitisation effect lasted for up to 3h after the return of hypoxia and was tumor specific as normal human astrocytes were not radiosensitised. Radiosensitisation was directly linked to the absence of HIF-1α in the nucleus, suggesting that the switch from glycolytic to mitochondrial energy metabolism was responsible for radiosensitisation [36], consistent with previous reports [8–13].

Hypoxia levels in different parts of a tumor fluctuate over time and in response to treatment. Accurately determining the level of tumor hypoxia is fundamental to the success of any hypoxia-modifying treatment in the clinical setting. This was demonstrated in the ARCON trial by the fact that only patients with hypoxic tumors benefited from treatment [32,37]. The use of external hypoxia probes, such as PIM used in the current study, requires tumour dissection and staining and as such give a snapshot of the levels of hypoxia of the tumour at that time. A direct, non-invasive measure of absolute tissue oxygenation is currently lacking. Positron emission tomography (PET) has been used with ^18^F- and ^64^Cu-labeled tracers that selectively locate in hypoxic tissues [38–40]. Thus, PET maps hypoxic cells, but does not report on the amount of oxygen dissolved in the tissue. Optical imaging methods demonstrate high sensitivity, but often suffer from very limited depth penetration. Bioluminescence imaging (BLI) has been used extensively in the last decade to image tumour growth in small animals [41] and can also serve to monitor hypoxia [42]. However, BLI requires the introduction of tumour cells transfected with luciferase and, like PET, reports on hypoxia, rather than providing a direct measure of tissue O_2_. Optical imaging techniques, including near infrared spectroscopy [43] and injectable oxygen-sensitive molecular probes [44] can be used to measure blood oxygen saturation and oxygen extraction fraction (NIRS) and oxygen dissolved in tissue (molecular probes). These methods, while powerful, are limited by the scattering of light as it travels through varying media (e.g., air, bone, tissue, and water) and, thus, are effective only in very superficial tissues and cell cultures. Magnetic resonance imaging (MRI) can be made sensitive to mesoscopic magnetic-field fluctuations surrounding blood vessels caused by changes in deoxyhemoglobin concentrations inside the vessel, the BOLD technique [45]. While complex biophysical models can be applied to calculate oxygen extraction fraction, BOLD MRI does not report directly on tissue pO_2_ [45–49]. Alternatively, methods have been developed recently to measure relative tissue pO_2_ [50,51] by exploiting the effect of O_2_, which is weakly paramagnetic, on ^1^H longitudinal magnetization relaxation.

The level of radiosensitisation achievable through removing hypoxia obviously depends on the initial level of tumor hypoxia. We saw an inconsistent low level of patchy hypoxia in intracranial GL261 tumors in mice compared to reproducibly robust levels of hypoxia in subcutaneous GL261 tumors, which has been described before [52]. Similar differences in hypoxia levels have been observed between subcutaneous and intracranial 9L rat gliomas [53], the latter containing a very small hypoxic fraction of 0–3% of tumor mass [22]. Intracranial C6 rat gliomas were significantly more hypoxic than GL261 and 9L gliomas [53]. Although the clinical relevance of animal models depends on the extent to which they mimic human tumours, the compromising effect of hypoxia on radiation efficacy is independent of the model used. The clinical relevance of our results therefore depends on the oxygenation levels of human GBM tumors. It is generally assumed that the level of hypoxia in human GBM tumors is severe and responsible to a large extent for treatment resistance. However, two detailed studies [41,42] measuring the levels of hypoxia in human GBMs *via* the binding of the 2-nitroimidazole EF5 [54] reported substantial inter-and intra-tumor heterogeneity of hypoxia in WHO grade 4 gliomas with the majority of cells being mild-to-moderately hypoxic (10%-0.5% pO_2_) rather than severely hypoxic (approximately 0.1% pO_2_). Similar to several glioma rodent models, the proportion of moderate to severely hypoxic cells was relatively low, even in high-grade gliomas. Both studies concluded that human brain tumors are dominated by normoxic to moderately hypoxic cells [41,42]. Although these clinical studies suggest that radiosensitisation by alleviating hypoxia may not be an optimal strategy for treating all high-grade gliomas, we believe that our radiosensitising results in intracranial tumors with inconsistent, minimal, patchy hypoxia warrant further research.
